# A Comparative Study of Fat Storage Quantitation in Nematode *Caenorhabditis elegans* Using Label and Label-Free Methods

**DOI:** 10.1371/journal.pone.0012810

**Published:** 2010-09-16

**Authors:** Kelvin Yen, Thuc T. Le, Ankita Bansal, Sri Devi Narasimhan, Ji-Xin Cheng, Heidi A. Tissenbaum

**Affiliations:** 1 Program in Gene Function and Expression, University of Massachusetts Medical School, Worcester, Massachusetts, United States of America; 2 Weldon School of Biomedical Engineering, Purdue University, West Lafayette, Indiana, United States of America; 3 Purdue Cancer Center, Purdue University, West Lafayette, Indiana, United States of America; 4 Department of Chemistry, Purdue University, West Lafayette, Indiana, United States of America; 5 Program in Molecular Medicine, University of Massachusetts Medical School, Worcester, Massachusetts, United States of America; Buck Institute for Age Research, United States of America

## Abstract

The nematode *Caenorhabditis elegans* has been employed as a model organism to study human obesity due to the conservation of the pathways that regulate energy metabolism. To assay for fat storage in *C. elegans*, a number of fat-soluble dyes have been employed including BODIPY, Nile Red, Oil Red O, and Sudan Black. However, dye-labeled assays produce results that often do not correlate with fat stores in *C. elegans*. An alternative label-free approach to analyze fat storage in *C. elegans* has recently been described with coherent anti-Stokes Raman scattering (CARS) microscopy. Here, we compare the performance of CARS microscopy with standard dye-labeled techniques and biochemical quantification to analyze fat storage in wild type *C. elegans* and with genetic mutations in the insulin/IGF-1 signaling pathway including the genes *daf-2* (insulin/IGF-1 receptor), *rict-1* (rictor) and *sgk-1* (serum glucocorticoid kinase). CARS imaging provides a direct measure of fat storage with unprecedented details including total fat stores as well as the size, number, and lipid-chain unsaturation of individual lipid droplets. In addition, CARS/TPEF imaging reveals a neutral lipid species that resides in both the hypodermis and the intestinal cells and an autofluorescent organelle that resides exclusively in the intestinal cells. Importantly, coherent addition of the CARS fields from the C-H abundant neutral lipid permits selective CARS imaging of the fat store, and further coupling of spontaneous Raman analysis provides unprecedented details including lipid-chain unsaturation of individual lipid droplets. We observe that although *daf-2*, *rict-1*, and *sgk-1* mutants affect insulin/IGF-1 signaling, they exhibit vastly different phenotypes in terms of neutral lipid and autofluorescent species. We find that CARS imaging gives quantification similar to standard biochemical triglyceride quantification. Further, we independently confirm that feeding worms with vital dyes does not lead to the staining of fat stores, but rather the sequestration of dyes in lysosome-related organelles. In contrast, fixative staining methods provide reproducible data but are prone to errors due to the interference of autofluorescent species and the non-specific staining of cellular structures other than fat stores. Importantly, both growth conditions and developmental stage should be considered when comparing methods of *C. elegans* lipid storage. Taken together, we confirm that CARS microscopy provides a direct, non-invasive, and label-free means to quantitatively analyze fat storage in living *C. elegans*.

## Introduction

One of the benefits of using *C. elegans* as a model organism is the ability to use powerful, high-throughput, forward genetic screens to discover important genes and pathways [1], [2]. Several papers have used these methods to determine genes that are involved in the accumulation of fat stores with possible applications to human obesity [3]–[5]. The regulatory roles of many of these genes are assigned based on dye-labeled assays for fat stores. However, dye-labeled assays for fat stores produce highly inconsistent results depending on the type of dyes or whether the staining is performed in live or fixed worms [6], [7]. Many dye-labeled assays for fat stores do not correlate with standard biochemical assays [6], [7]. Consequently, the functional assignments of many genes related to fat storage based on a single method may not be accurate and potentially hinders the functional studies of genes that control fat accumulation in *C. elegans*.

In recent years, an alternative to dye-labeled assay to quantify fat stores in *C. elegans* has been described with coherent anti-Stokes Raman scattering (CARS) microscopy [8], [9]. CARS microscopy is a label-free chemical imaging technique that relies on intrinsic molecular vibration as a contrast mechanism [10], [11]. CARS microscopy allows direct visualization of lipid-rich organelles due to the abundance of the CH_2_ group that has the symmetric stretch vibration frequency of 2840 cm^−1^. In addition, CARS microscopy is highly multifunctional. It is capable of simultaneous chemical and fluorescent imaging, which allows label-free visualization of both neutral lipid droplets and autofluorescent granules [9]. Furthermore, CARS is capable of Raman spectral analysis, which allows assaying of lipid-chain unsaturation and lipid packing order of individual lipid droplets [8], [9], [12]–[14]. With such versatility, CARS microscopy has the potential to become a robust and reliable method to screen for the function of genes that control lipid metabolism in living *C. elegans*.

However, technical challenges are hindering the widespread adoption of CARS microscopy for *C. elegans* research. First, the very expensive price tag for a CARS microscope places it beyond the affordability of most researchers. Secondly, setting up a CARS microscope system requires expertise in nonlinear optics, which may be a challenge to many biologists. Thirdly, dye-labeled assays are inexpensive and readily available which render them attractive choices**.** And lastly, many biologists are unfamiliar with the capability of CARS and other nonlinear optical imaging modalities.

**Figure 1:**
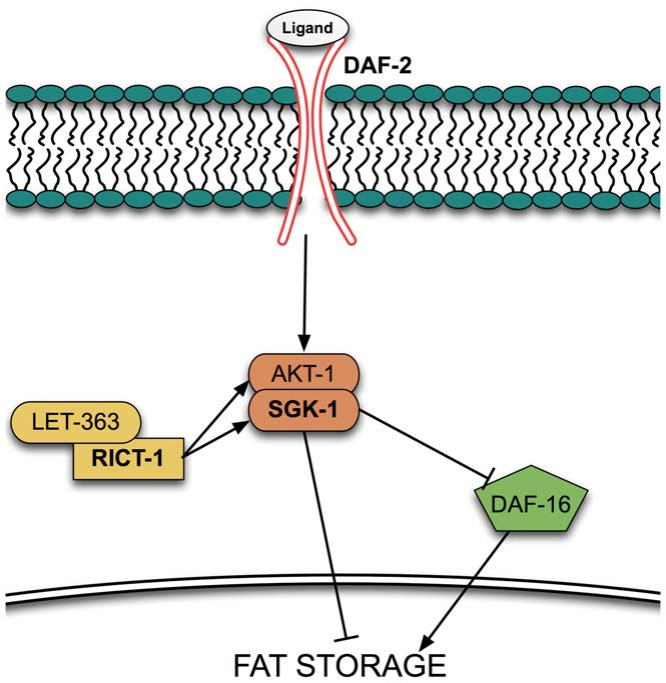
Simplified diagram of insulin/IGF signaling. Bolded proteins are proteins that have been investigated in this study.

In this paper, we explore the capability of CARS microscopy for quantitative analysis of fat storage in wild type worms and three strains with mutations in genes involved in the insulin/IGF-1 signaling pathway: *daf-2(e1370), rict-1(ft7),* and *sgk-1(ok538)* (**Figure 1**). We directly compare the abilities of CARS with several dyes commonly used in *C. elegans,* as well as triglyceride quantification to study fat storage. Each of these three mutants has been reported to have abnormal fat stores compared to wild type using a variety of techniques [6], [7], [15]. *daf-2(e1370)* mutants bear a mutation in the insulin/IGF receptor [15], while *rict-1* mutants have a mutation in the RICTOR (Rapamycin Insensitive Companion of mTOR) gene [16], [17]. *sgk-1* mutants have a mutation in the serum/glucocorticoid regulated kinase (SGK) gene [18]. Previous studies using vital stains to assay for fat stores has produced variable results that are inconsistent with standard biochemical assays [6], [7]. We compare the performance of label-free CARS imaging to both dye-labeled imaging and triglyceride quantification to further investigate quantitation of fat stores in *C. elegans*. We aim to introduce CARS microscopy to researchers who are unfamiliar with its capability for lipid studies in *C. elegans*. Using CARS, we are able to identify the sources of errors in dye-labeled assays. We also stress the importance of similar growth conditions as well as similar developmental stages when trying to compare methods. Therefore, these studies aim to help researchers improve the accuracy for fat store measurements by standard dye assays should CARS microscopy remain inaccessible.

## Results

### CARS/TPEF imaging reveals co-existing neutral lipid stores and autofluorescent granules in *C. elegans*

**Figure 2:**
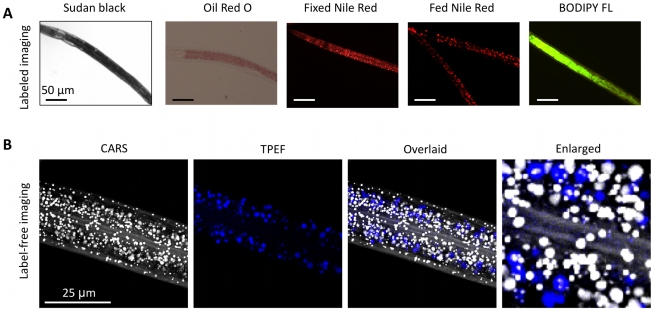
Label and label-free imaging methods to assay fat storage in *C. elegans*. (**A**) Labeled imaging of fat stores using Sudan Black, Oil Red O, and Nile Red staining of fixed worms and Nile Red and BODIPY-labeled fatty acids fed to live worms. (**B**) Label-free visualization of neutral lipid species and autofluorescent gut granules using simultaneous CARS and TPEF imaging, respectively. Note the co-localization of TPEF signal with CARS signal in the intestinal cells. Images are presented as 3-D stacks of 30 frames taken at 1 µm increment along the vertical axis. Rightmost panel is an enlargement of the overlaid image with the xy dimensions of 20 µm×20 µm. It should be noted that the association of fluorescent signal with lipid signal can be found surrounding, at one end, above, or below the lipid signal. In addition, the lipid contents of the fluorescent particles vary from one to another. For the particular image presented, the fluorescent puncta do exhibit lipid signal when examined at a higher magnification.

Fat stores in *C. elegans* can be visualized by dye-labeled imaging or by label-free imaging (**Figure 2** **, Figure S1, S2**). Dye-labeled imaging of fat stores relies on the use of fat-soluble dyes such as Sudan Black, Oil Red O, Nile Red, or BODIPY (**Figure 2A** **, Figure S1, S2**). Sudan Black and Oil Red O are fixative-based dyes, whereas Nile Red and BODIPY are vital dyes, which are fed to live worms to assay fat stores [3], [7], [15], [19]. In contrast, label-free imaging of fat stores with CARS microscopy relies on the intrinsic molecular vibration of CH_2_ groups for the contrast mechanism (**Figure 2B**). Simultaneous CARS and two-photon excited fluorescent (TPEF) imaging allows label-free visualization of both neutral lipid droplets and autofluorescent gut granules [9]. Autofluorescent gut granules have been classified as lysosome-related organelles (LROs) whose expression increases with aging and oxidative stress [20], [21]. The partial overlap of the TPEF and CARS signals (**Figure 2B**) is possibly due to the insufficient depth resolution of our technique. We have observed that all autofluorescent granules contain some CARS signal although the intensity varies from granule to granule. However the converse is not true and all CARS signal is not associated with autofluorescent granules. Thus, multimodal CARS/TPEF imaging suggests the co-existence of 1) neutral lipid stores that resides in both the hypodermis and the intestinal cells, and 2) autofluorescent organelles that reside exclusively in the intestinal cells.

### Quantitative measurements of fat stores in living *C. elegans*

**Figure 3:**
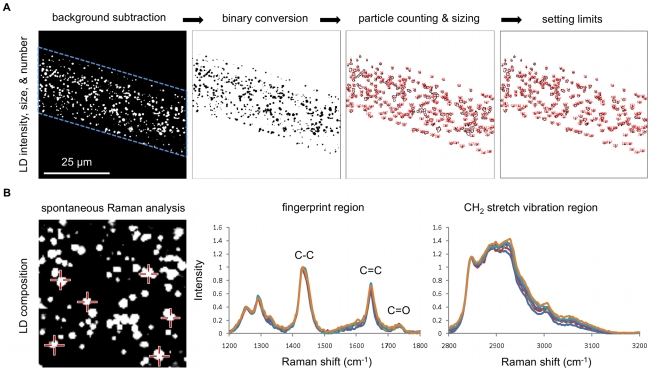
Quantitative analysis of the total fat stores and the size, number, and composition of lipid droplets with CARS microscopy. (**A**) Total fat stores are defined as the integrated CARS signal intensity over the probed volume minus the background signal arising from worm bodies (leftmost panel). The ImageJ software is used for binary image conversion of CARS images and for particle analysis to determine the size and number of lipid droplets. Upper limits (2 µm^2^) can be manually set to minimize sizing errors due to lipid droplets being too close to each other. (**B**) The compositions of six lipid droplets (crosshairs) are analyzed using spontaneous Raman spectroscopy. Statistically insignificant variability in lipid composition is observed for lipid droplets within a single worm. Leftmost panel is an enlargement of Figure 2A with the xy dimensions of 20 µm×20 µm.

CARS microscopy allows quantitative measurements of fat stores in living *C. elegans*. The CARS signal typically arises from both the cytoplasmic lipid droplets or fat stores and the phospholipid membranes (**Figure 2B**). Importantly, the coherent addition of CARS fields produces a signal from the C-H abundant lipid droplets much larger than that from other cellular organelles. To quantify the fat stores, a threshold can be set such that the CARS signal from the phospholipid membrane and other cellular structures is removed. Integration of the remaining CARS signal intensity yields a numerical value for total fat stores of the probed volume (**Figure 3A**). In addition, a CARS image can be converted to a binary image for measurements of number and size distribution of lipid droplets using the particle analysis function of the ImageJ software. This approach provides a robust means for lipid droplet analysis. However, when the lipid droplets are too close to one another, errors can be introduced. In such a case, limits can be set for additional optimization of the quantitation (**Figure 3A**). Furthermore, spontaneous Raman spectral analysis can provide critical information on the lipid chain of the lipid droplets (**Figure 3B**). The ratio of C = C over C-C or I_1660_/I_1445_ is a reliable measure of lipid-chain unsaturation and the ratio of asymmetric to symmetric CH_2_ stretch vibration or I_2880_/I_2845_ can be used to measure lipid-chain packing order [12]. Taken together, CARS microscopy provides quantitative measurements of single lipid droplets that have not been described with standard dye-labeled assays.

### Comparison of CARS imaging with dyes in insulin/IGF-1 signaling mutants

**Figure 4:**
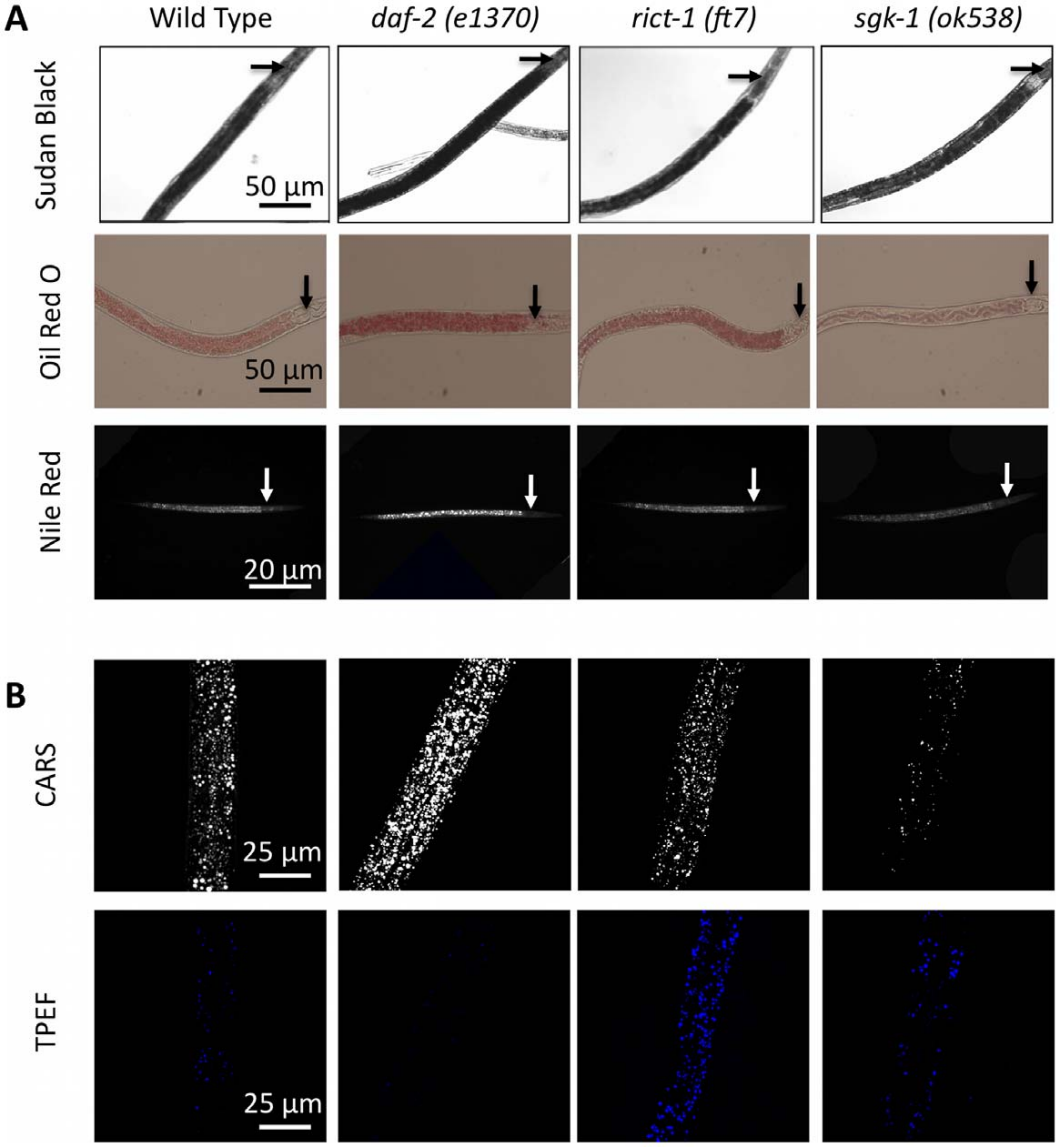
Label and label-free imaging of fat storage in wild type and mutant *C. elegans*. (**A**) Visualization of fat stores in fixed worms using fixative dyes Sudan Black, Oil Red O, and Nile Red*. Arrows indicate the pharynx (**B**) Visualization of fat stores (upper panels) and autofluorescent gut granules (lower panels) using simultaneous CARS and TPEF imaging of living worms, respectively. Images are presented as 3-D stacks of 30 frames taken at 1 µm increment along the vertical axis. *Nile Red pictures were taken with a more sensitive black and white camera.

To evaluate the performance of CARS microscopy, we compare CARS imaging assays with standard dye-labeled assays to quantitate fat stores. We chose three mutants that appeared to have different lipid profiles by CARS that also happen to affect insulin/IGF signaling (**Figure 1**). We assessed lipid stores in all of the three mutants in comparison to wild type. As shown in Figure 3, we find that staining fat stores in fixed worms with fixative dyes Oil Red O and Sudan Black produce results that partially correlate with CARS imaging of living worms. Qualitatively, we observe that *daf-2* mutants exhibit a significant increase in staining level, *rict-1* worms exhibit a moderate increase in staining level, and *sgk-1* worms exhibit a reduction in staining level as compared to wild type worms (**Figure 4A** **, Figure S1, S2 and quantified in Figure S3**). Notably, both fixative dyes give similar results. Similarly, label-free CARS imaging also shows increased fat stores in *daf-2* mutants and reduced fat stores in *sgk-1* worms as compared to wild type worms (**Figure 4B**). Thus, the data from the two fixative stains, Oil Red O and Sudan Black, agree with CARS imaging data on *daf-2* and *sgk-1* worms. However, CARS imaging shows that *rict-1* mutants have similar lipid stores when compared to wild type, which is in contrast to the dye staining.

To further investigate these differences, we used additional analysis with CARS imaging. When we used simultaneous TPEF imaging, we observed that the level of autofluorescent granules is lower in *daf-2* mutants, higher in *rict-1* mutants, and similar in *sgk-1* mutants when compared with wild type worms (**Figure 4B**). However, the autofluorescent phenotypes of these mutants have largely been omitted in the literature. It is possible that the dramatic increase in the level of autofluorescent species in *rict-1* worms could interfere with the evaluation of neutral lipid species using fixative dyes.

### Fed Nile Red and Fed BODIPY do not reveal fat stores

**Figure 5:**
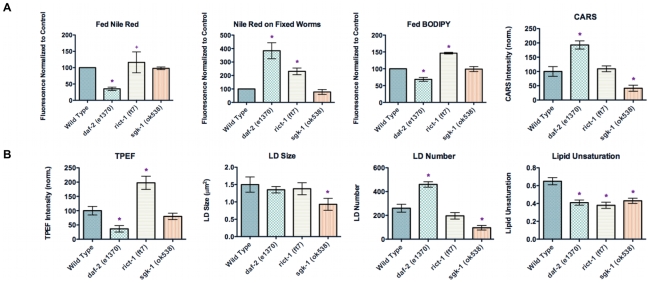
Quantitative analysis of fat storage in wild type and mutant *C. elegans*. (**A**) Total fat stores in mutant worms relative to wild type worms assayed by feeding worms with vital dyes Nile Red and BODIPY-labeled fatty acids, with CARS imaging, and with fixative staining with Nile Red. Data represent the average of 3 independent trials with an average of 18 worms quantified per trial for fed Nile Red, 20 worms quantified per trial for fed BODIPY, 9 worms quantified per trial for CARS imaging, and 23 worms quantified per trial for Nile Red on fixed worms. Data are normalized to 100 for wild type worms and comparatively for mutant worms. Error bars represent the standard error. (**B**) Quantitative analysis of autofluorescent granules using TPEF imaging and size, number, and lipid unsaturation of lipid droplets using CARS imaging. TPEF data are normalized to 100 for wild type worms and comparatively for mutant worms. Lipid unsaturation represents the ratio of C = C peak intensity over C-C peak intensity, or I_1660_/I_1445_. Data represent the average of 3 independent trials with lipid droplets of 9 worms quantified per trial, or 27 worms total. Error bars represent the standard error.

We next examined staining fat stores by feeding worms with vital dyes Nile Red and BODIPY. As shown in Figure 5, the results from feeding worms dyes to stain lipids produce data that do not correlate with CARS imaging of live worms. Quantifying the total fluorescence from the worms using ImageJ from vital labeling show reduced fat stores in *daf-2* worms, increased fat stores in *rict-1* worms, and similar fat stores in *sgk-1* worms as compared to wild type worms (**Figure 5A**). We find that the Nile Red and BIODIPY vital labeling data do not agree with fixative labeling data using Oil Red O, Sudan Black, and Nile Red, or with label-free CARS imaging data. Our results show that there is an intrinsic difference between the capacities of dyes to stain fat stores in live and fixed worms. Previously, Nile Red staining of fixed worms has been shown to be a better proxy for fat stores than Nile Red staining of live worms [6]. Indeed, our assays for fat stores using Nile Red staining of fixed *daf-2* and *sgk-1* worms produce data that in general tend to agree with those obtained with fixative dyes and CARS imaging (**Figure 5A**). However, fixed Nile Red staining also shows an increase in fat stores in *rict-1* worms, whereas, CARS imaging show comparable level of fat stores relative to wild type worms. Further verification of fat stores using biochemical methods corroborates the CARS findings (**see Text S1 and Figure S4**).

### Quantitation of lipids with CARS

Next, we further extended our analysis of lipids in *C. elegans* using CARS imaging. In addition to assaying total fat stores, CARS imaging allows quantitative analysis of autofluorescent granules, size and number of lipid droplets, and the degree of lipid-chain unsaturation (**Figure 5B**). Compared to wild type worms, *daf-2* mutants exhibit a two-fold increase in the CARS signal and a three-fold reduction in the autofluorescent signal. *rict-1* mutants when compared to wild type, exhibit similar levels of the CARS signal and a two-fold increase in the autofluorescent signal. *sgk-1* mutants exhibit a three-fold decrease in the CARS signal and a twenty percent reduction in the autofluorescent signal when compared to wild type. When we examined the size of lipid droplets, we found that the lipid droplet size is approximately 1.4 µm^2^ in wild type worms as well as *daf-2* and *rict-1* mutants, while *sgk-1* mutants were approximately 1 µm^2^. Further analysis showed that compared to wild type worms, *daf-2* mutants have a two-fold increase in the number of lipid droplets and *sgk-1* mutants have one-third reduction in lipid droplets (**Figure 5B**). The number of lipid droplets in *rict-1* mutants is statistically comparable to that in wild type worms. In general, all three mutants have a lower degree of lipid-chain unsaturation compared to wild type worms. One possibility for this lower lipid-chain unsaturation is that mutation in either *daf-2*, *rict-1*, or *sgk-1* might have a negative effect on the expression or activity of lipid desaturation enzymes, or the ability to uptake unsaturated dietary lipids.

### Examining sources of errors with fat storage analysis in *C. elegans*

**Figure 6:**
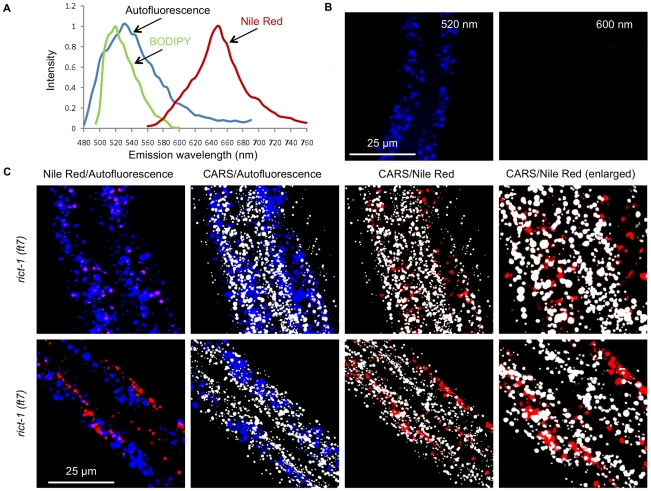
Possible errors in dye-labeled assays to analyze fat stores in living *C. elegans*. (**A**) Emission spectra of autofluorescent granules, BODIPY, and Nile Red obtained with microspectrometry. The autofluorescent granules and BODIPY are excited at 457 nm using an Argon-Ion continuous-wave laser. The Nile Red is excited with a 543 nm Helium-Neon continuous-wave laser. (**B**) Insignificant bleed-through of autofluorescent granule signal is observed using a 600 nm bandpass filter and two-photon excitation at 885 nm. (**C**) CARS and TPEF imaging of two *rict-1* worms fed with Nile Red. Autofluorescent granules (blue) are detected with a 520 nm bandpass filter and Nile Red (red) is detected with a 600 nm bandpass filter. Pink color is the result of overlapping blue and red colors which indicates co-localization of autofluorescent granules and Nile Red. Note the complete co-localization of autofluorescent granules and Nile Red in worm 1 (upper panels) and partial co-localization of autofluorescent granules and Nile Red in worm 2 (lower panels). Rightmost panels are enlargements of the CARS/Nile Red overlaid images with the xy dimensions of 20 µm×20 µm. Note the poor co-localization of lipid droplets (grey) and Nile Red (red). Images are presented as 3-D stacks of 30 frames taken at 1 µm increment along the vertical axis.

CARS microscopy measures fat stores directly, whereas fixative or vital dyes serve as proxies for fat stores. Therefore, the inconsistencies between dye-labeled assays are likely due to labeling efficiency or errors during quantitation of the dye signal. To try to investigate the source of errors in dye-labeled assays, we first examined the spectral properties of two fluorescent dyes, Nile Red and BODIPY as well as the autofluorescent granules. We find that the emission spectra of autofluorescent granules and BODIPY peak at 530 nm and 520 nm, respectively, when excited with a 457 nm continuous-wave laser (**Figure 6A**). The broad emission spectrum of the autofluorescent granules therefore suggests that when fat stores are assayed with BODIPY, the readout inevitably includes the autofluorescent signal. Furthermore, a recent study shows that BODIPY does not stain fat stores when fed to live worms [7]. Taken together, BODIPY should not be used as a proxy for fat stores in live or fixed worms.

On the other hand, the conditions for Nile Red signal detection vary among researchers. Some researchers used the Rhodamine emission filters that centers at 575 nm to detect the Nile Red signal [3]. At this wavelength, significant autofluorescent signal can still be detected according to the spectral data (**Figure 6A**). Thus, some errors in the Nile Red assays for fat stores can be attributed to the signal bleed-through from the autofluorescent granules. Ideally, the emission filter for Nile Red should center around 650 nm. Nonetheless, we observe minimal bleed-through of the autofluorescent signal using a 600 nm emission filter and two-photon excitation at 885 nm (**Figure 6B**). In fact, most researchers use the emission filters that center around 600 nm, yet vital staining of fat stores using Nile Red still does not correlate with biochemical assays [5]–[7].

To further investigate the source of errors by obtaining CARS and fluorescent images of live worms fed with Nile Red dye, we obtained fluorescent images at both 520 nm for autofluorescent granules and 600 nm for Nile Red dye (**Figure 6C**). We observed that in some worms Nile Red signal completely co-localizes with the autofluorescent signal (**Figure 6C** **top row)**. This observation is consistent with many previous findings where autofluorescent granules were identified as LROs and Nile Red was reported to stain LROs [7], [20]. The presence of more autofluorescent signal than the Nile Red signal could be due to insufficient Nile Red concentration or due to the degradation of the Nile Red dye. In some other worms, only some of the Nile Red signal co-localizes with the autofluorescent signal (**Figure 6C** **bottom row**). However, in all worms, the Nile Red signal localizes poorly with the CARS signal for fat stores. This observation is quite surprising given the proven ability of Nile Red to stain lipid droplets in mammalian cells and in fixed worms [6], [22]. It is highly probable that Nile Red enters the intestinal cells of *C. elegans* via endocytosis, and then remains in the endosomes which eventually mature into lysosomes [20]. It is unlikely that Nile Red enters the cytoplasm where the lipid droplets reside because in this scenario Nile Red should stain lipid droplets given its nonspecific affinity for lipids in general. Our data clearly suggests that Nile Red should not be used as a proxy for fat stores in live worms.

## Discussion

As *C. elegans* is increasingly used as a model system to study the basic mechanisms of fat storage and energy homeostasis, it is critical to ensure that the established assays accurately determine and reflect fat content. To that effect, here we study the performance of existing techniques including vital and fixative labeling and label-free assays to evaluate fat stores. We find that vital staining with the fat-soluble dyes Nile Red and BODIPY labeled fatty acids cannot serve as proxies for fat stores. Nile Red and BODIPY-labeled fatty acids do not stain fat stores but stain autofluorescent organelles previously identified as LROs in living worms [7]. It is possible that Nile red and BODIPY-labeled fatty acids are actively transported into intestinal cells. In fact, a specific transporter for Nile Red has been identified in yeast [23]. Similar transporters in worms may sequester Nile Red and BODIPY-labeled fatty acids, thus shielding them from the fat stores in living worms. Furthermore, Clokey and Jacobson reported over two decades ago that exogenous fluorescent probes are taken up by endocytosis and accumulate within the autofluorescent lipofuscin granules, which are the secondary lysosomes and active recipients of endocytosed fluorescent probes [20]. Our findings, together with other independent reports, strongly suggest that vital staining using fat-soluble dyes including both Nile Red and BODIPY, should not be used to assay fat stores in living *C. elegans*.

We find that fixative staining using Sudan Black, Oil Red O, and Nile Red produce consistent and reproducible data on fat stores (**Table 1**). Qualitatively, fixative staining data agree with CARS imaging data on the level of fat stores in mutant *daf-2* and *sgk-1* relative to wild type worms. However, our fixative staining data differ with CARS imaging data and biochemical analysis on the level of fat stores in *rict-1* mutants where there is a high level of autofluorescent gut granules/lipofuscin. These results are in contrast to those found by Soukas *et al.* [16] and are further discussed in Text S1. Since CARS microscopy provides a direct measure of fat stores instead of a proxy of fat storage, it is possible that this difference could be attributable to errors introduced during fixation or staining procedures. Additional error could also be due to the following reasons. First, lipofuscin is a hydrophobic accumulation of highly oxidized cellular proteins and lipids that have characteristic fluorescent properties [24], [25]. Their hydrophobic properties pose a problem for lipophilic stains because both Sudan Black and Oil Red O stain for lipofuscin as well as triglycerides [26], [27]. As Nile Red is also a hydrophilic stain, it is possible that it could also stain lipofuscin. Therefore, fixative staining cannot distinguish neutral fat stores from lipofuscin. Second, the autofluorescence of lipofuscin might contribute to errors in the quantitation of fluorescent stains such as Nile Red and BODIPY. Third, formaldehyde fixation of the worms introduces additional non-specific fluorescence due to the fluorescent properties of the Schiff bases produced [28]. Finally, non-specific staining of dyes, variable dye concentration, and thoroughness of washing procedures are among the other factors that might contribute to the inaccuracy in assaying for fat stores using labeling techniques [29]–[31]. Therefore, although fixative staining offers a simple and reproducible means to assay fat stores in *C. elegans*, we suggest that errors may arise due to interference from autofluorescence granules and non-specific staining of lipofuscin and other cellular organelles. Thus, fixative staining to assay for fat stores in *C. elegans* should also present data on autofluorescent granules.

**Table 1:** Qualitative comparison of fat stores in mutants relative to wild type worms.

	Strain		
Method	*daf-2 (e1370)*	*rict-1 (ft7)*	*sgk-1 (ok538)*
Fed Nile Red	decrease	increase	no change
Fed BODIPY fatty acids	decrease	no change	no change
Fixed Nile Red	increase	increase	decrease
Fixed Sudan Black	increase	increase	decrease
Fixed Oil-Red-O	increase	increase	decrease
CARS (neutral lipids)	increase	no change	decrease
CARS (autofluorescence)	decrease	increase	no change

In contrast to labeling techniques, label-free CARS imaging provides a direct and reliable means to assay fat stores in living *C. elegans*. In addition, CARS imaging allows for detailed analysis of the number, size, and lipid-chain unsaturation of single lipid droplets. Multimodal CARS and TPEF imaging further allows simultaneous visualization of neutral lipids and autofluorescent lipofuscins. Together, CARS microscopy allows for the assaying of various aspects of fat storage in living *C. elegans* in a non-invasive and label-free manner. Fat storage in the form of lipid droplets has recently emerged as a complex process that is dependent on insulin signaling, phospholipid synthesis, fatty acids synthesis and desaturation, fatty acids uptake and transport, activation of nuclear hormone receptors, and upregulation of a wide range of lipid synthesis enzymes [32]–[39]. Genetic mutations of genes that control lipid metabolism in *C. elegans* and other organisms have been shown to impact the total fat stores, as well as the size, number, and composition of lipid droplets [36], [40], [41]. Furthermore, a strong correlation between fat storage, oxidative stress, and the lifespan of *C. elegans* has been revealed in recent years [15]–[17], [42]–[44]. Therefore, the versatility of CARS microscopy is critical not only for forward genotype-phenotype screening of genes that control fat accumulation, but also for the studies of potential interactions of lipid metabolism genes. Most importantly, CARS microscopy is currently the only reliable method to assay for fat stores in living *C. elegans*. When combined with recent advances in microfluidic devices [45]–[47], this capability should allow dynamic studies of the correlation between lipid metabolism, behavioral response, and aging over the lifetime of a single worm.

### Conclusions

We have used several methods including fixative stains, live stains and live imaging to analyze *C. elegans* fat storage. Our data supports previous publications that vital dyes should be used with caution for lipid analysis in *C. elegans*. Using CARS microscopy we show Nile Red itself stains the autofluorescent granules and this is the confounding problem. Importantly, we show that similar stages and growth conditions should be used when comparing different methods (**Figure S5**). We suggest that CARS is the optimal method for *C. elegans* fat storage. However, when not available, we suggest using one of the fixative methods as well as checking autofluorescent levels.

## Materials and Methods

### Worm strains and growing conditions

All *C. elegans* strains used in this work were obtained from the *Caenorhabditis* Genetics Center. Wild type N2, *daf-2(e1370), sgk-1(ok538)* and *rict-1(ft7)* mutants were maintained at 15°C using standard *C. elegans* techniques [48]. All strains except for *daf-2(e1370)* were shifted to room temperature at 22.5°C overnight, while *daf-2(e1370)* worms were shifted to 20°C overnight, prior to label or label-free assays. All assays were repeated for a total of three times using worms at the L3/L4 stage.

### Worm fixation

Worms were washed twice with 1 x PBS and then suspended in 120 µl of PBS to which an equal volume of 2X MRWB buffer (160 mM KCl, 40 mM NaCl, 14 mM Na_2_EGTA, PIPES pH 7.4, 1 mM Spermidine, 0.4 mM Spermine, 30 mM, 2% Paraformaldehyde, 0.2% beta-mercaptoethanol) was added. The worms were taken through 3 freeze-thaw cycles between dry ice/ethanol and warm running tap water, followed by spinning at 14000g washing once in PBS to remove paraformaldehyde.

### Nile Red and BODIPY-labeled fatty acid feeding protocol

Nile Red (Cat. No. N1142, Invitrogen, Carlsbad, CA, USA) diluted in water (100 ng/mL final concentration) or BODIPY-labeled fatty acids (Cat. No. D-3823, Invitrogen, Carlsbad, CA, USA) diluted in water (1 µM final concentration) was overlaid on an NGM media plate and allowed to dry overnight. Worms were then transferred to the plate for at least 24 hours before fixing the worms.

### Fixed Nile Red Staining

A stock solution was made by dissolving Nile Red in acetone. It was then diluted in water (100 ng/mL final concentration) and fixed worms were incubated overnight in the working solution.

### Sudan Black Staining

Sudan Black (Cat. No. 199664, Sigma-Aldrich, St. Louis, MO, USA) staining of stored fat was performed as previously described [15], except for the fixation step as described above. After fixation, worms were sequentially dehydrated by washes in 25%, 50% and 70% ethanol. Saturated Sudan Black solution was prepared fresh in 70% ethanol. The fixed worms were incubated overnight in 250 µL of Sudan Black solution, on a shaker at room temperature.

### Quantification of Sudan Black staining

Using ImageJ, we measured the average pixel intensity for a 30-pixel radius immediately behind the pharynx of each animal. A minimum of 9 animals was measured for each strain and we repeated the experiments an additional 2 times. Significance was determined by Student's t-test.

### Oil Red O Staining

Oil Red O staining was performed as previously described [16]. After fixation, worms were resuspended and dehydrated in 60% isopropanol. Approximately 250 µL of 60% Oil Red O stain (Cat. No. O9755, Sigma-Aldrich, St. Louis, MO, USA) was added to each sample, and samples were incubated overnight at room temperature. Oil Red O was prepared as follows: 0.5 g of Oil Red O powder was dissolved in 100 ml isopropanol solution and equilibrated for several days. The solution was then freshly diluted with 40% water to get a 60% stock and allowed to sit 10 minutes at room temperature and filtered using 0.2 to 0.4 mm filters.

### Quantification of Oil Red O staining

Using ImageJ, we separated out each color image into its RGB channel components. As it has been reported that Oil Red O absorbs light at 510 nm, we used the green channel for further analysis [49]. We measured the average pixel intensity for a 40 pixel radius immediately behind the pharynx of each animal. In addition, we measured a 40 pixel radius of the background, which was later subtracted from the values obtained from the staining. A minimum of 9 animals was measured for each strain and we repeated the experiments an additional 2 times. Significance was determined by Student's t-test.

### Triglyceride Assay

Biovision's Triglyceride Quantification Kit (Mountain View, CA) was used to assay for triglyceride content. Worms were synchronized by hypochlorite treatment and then grown at 15°C. They were then upshifted to 20°C for 8 hours until they were at the L3/L4 stage. Worms were then collected and washed with S basal solution. A 5% Triton X-100 solution with 1x protease inhibitors (Roche cOmplete mini, EDTA-free Indianapolis, IN) was added 1∶1 to a 50 uL worm pellet. The worms were sonicated with a Bioruptor (Diagenode, Sparta, NJ) using power output 4 for 10 seconds. Protein content was estimated by BCA method. Lipids were dissolved by heating the lysate to 90°C for 5 minutes followed by vortexing. This was preformed twice before the lysate was cleared by centrifugation. The supernatant was then used for the triglyceride assay per the manufacturers instructions. At least 2 biological replicates were used for each strain and three technical replicates were used for each biological replicate.

### Imaging conditions for dye-labeled assays

Fixed worms were mounted on slides and visualized using a Zeiss Axioscope 2+ microscope. ImageJ software was used to quantify total fluorescence from pictures obtained from the microscope. Exposure times were kept constant within each trial. NR and SB images were obtained using an ORCA ER CCD camera (Hamamatsu Photonics, Japan). ORO images were obtained using an Axiocam HRc CCD camera (Carl Zeiss, Thornwood, NY). Fluorescent dyes were excited using a 100W HBO mercury lamp in conjunction with a FITC (480ex/535em) or TRITC (540ex/605em) filter set (Chroma Technology, Bellows Falls, VT) for BODIPY or NR respectively.

### A multifunctional CARS microscope

Two mode-locked 5-ps Ti:sapphire lasers (Tsunami, Spectra-Physics, Mountain View, CA) were synchronized to each other through an electronic module controller (Lok-to-Clock, Spectra-Physics). The two parallel-polarized laser beams, pump and Stokes, were collinearly combined and sent into a laser scanning confocal microscope (FV300/IX71, Olympus America, Center Valley, PA). Pump and Stokes lasers were tuned to 14140 cm^−1^ (or 707 nm) and 11300 cm^−1^ (or 885 nm), respectively, to be in resonance with the CH_2_ symmetric stretch vibration at 2840 cm^−1^. The combined beams were focused into the sample through a 60x water immersion microscope objective with a 1.2 numerical aperture. Forward-detected CARS signal was collected by an air condenser with a 0.55 numerical aperture, transmitted through a 600/65 nm bandpass filter (Cat. No. 42-7366, Ealing Catalog, Rocklin, CA), and detected by a photomultiplier tube (PMT, H7422-40, Hamamatsu, Japan). Simultaneously, back-reflected TPEF signal was collected by the same illuminating objective, spectrally separated from the excitation source, transmitted through a 520/40 nm bandpass filter (Cat. No. 42002, Chroma Technology, Bellows Falls, VT), and detected by a photomultiplier tube (PMT, H7422-40, Hamamatsu, Japan) mounted at the back port of the microscope. To detect Nile Red fluorescence, the pump beam was blocked and the Stokes beam at 885 nm was used for excitation. To obtain Raman spectra of lipid droplets, the Stokes beam was blocked and the pump-laser induced Raman scattering signal was directed toward a spectrometer to permit spectral analysis from 830 cm^−1^ to 3100 cm^−1^, which covers both the fingerprint and the CH-stretch vibration regions. A spectrometer with a 300 g/mm grating and a TE cooled back-illuminated EMCCD (Newton 920-BRD, Andor Technology, Belfast, Ireland) was mounted to the side port of the microscope to allow spontaneous Raman spectral analysis on the same microscope platform. Average acquisition time for a 512×512 pixels CARS image was 1.12 second and a full-spectral Raman analysis was 4 seconds. The combined Stokes and pump laser power was kept constantly at 40 mW. For all Raman spectral measurements, pump laser power was reduced to 10 mW.

### CARS imaging conditions and data analysis

All *C. elegans* were anesthetized in a droplet of 100 mM sodium azide and mounted on fresh 2% agarose slides prior to imaging. To evaluate the expression level of neutral and autofluorescent bodies, a probe volume with xyz dimensions of 125 µm×125 µm×29 µm was defined at the mid-section of wild type or mutant worms. Simultaneous depth imaging with CARS and TPEF along the vertical (z) axis was performed at 1 µm step size to obtain 30 frames. Total CARS and TPEF signal arising from worms were integrated over 30 frames and divided by the worm volumes to obtain average pixel intensity values. Thus, the average pixel intensity values were not affected by size variability among worms. Background CARS and TPEF signal were defined as those arising from the worm bodies devoid of lipid droplets or autofluorescent granules. Background CARS and TPEF pixel intensity were subtracted from the average pixel intensity of CARS and TPEF signal to obtain CARS signal for fat stores and TPEF signal for autofluorescent granules, respectively. Fluoview software (Olympus America, Center Valley, PA) was used for image acquisition and display. ImageJ software was used for particle analysis.

## Supporting Information

Text S1 (0.03 MB DOC)

Figure S1 Additional Sudan Black stained worms. Arrow indicates the pharynx of the worm.
(2.28 MB TIF)

Figure S2 Additional Oil Red O stained worms. Arrow indicates the pharynx of the worm.
(3.65 MB TIF)

Figure S3 Quantification of Sudan Black and Oil Red O staining. Both daf-2 and rict-1 have increased staining compared to wild type. sgk-1 mutants have decreased staining. * indicates a significant difference compared to wild type. + in 2 out of 3 trials there was a significant difference from wild type.
(1.44 MB TIF)

Figure S4 Quantification of triglyceride stores. Corroborating the data from the CARS quantification, there is a significant increase in triglycerides in daf-2 worms, a significant decrease in triglycerides in sgk-1 worms, and no change in triglycerides in rict-1 worms. Triglycerides has been normalized to total protein levels and the units for the Y-axis is nmoles of triglycerides/gram of protein.
(0.49 MB TIF)

Figure S5 Differential fat stores in larval stage 3 (L3) worms compared to larval stage 4 (L4) worms. Typical Oil Red O staining of an L3 sgk-1 worm compared to an L4 sgk-1 worm. The top panel is an L3 worm and the bottom is an L4 worm.
(7.78 MB TIF)
