# Supplementary material for: A Comparative Study of Fat Storage Quantitation in Nematode Caenorhabditis elegans Using Label and Label-Free Methods
Source: PLoS One. 2010 Sep 16;5(9):e12810. doi: 10.1371/journal.pone.0012810 (PMC2940797; doi:10.1371/journal.pone.0012810)
Supplement: Text S1 — (0.03 MB DOC) [file pone.0012810.s001.doc]

**Supplemental Results**

Quantification of triglycerides was performed using a triglyceride assay kit (Biovision, Mountain View, CA) and normalized to total protein as determined by bicinchoninic acid (BCA) assay (**Figure S4)**. In agreement with the CARS and fixed staining data, *daf-2* worms showed a significant increase in triglyceride amount and *sgk-1* worms had a significant decrease. In contrast, *rict-1* worms did not have a significant change in triglyceride levels, supporting the CARS data.

**Supplemental Discussion**

The results of the triglyceride quantitation agree with our CARS studies. Compared to wild type, we see that *daf-2* mutants have a significant increase in fat, *sgk-1* mutants have a significant decrease, and *rict-1* mutants are not significantly different. These data appear to be in contrast to the results obtained by Soukas *et al.* where *rict-1* mutants showed a significant increase in the ratio of triglycerides to phospholipids [16]. There are several factors that could account for these differences including the developmental stage of the worm, the temperature at which the worms were kept, and the specific allele that causes the mutation, all of which were different between the two studies. We have found that the developmental stage of the worm can cause a significant difference in staining (**Figure S5**). Moreover, temperature is known to affect mutants of the insulin/IGF pathway and the growth conditions must be considered when comparing lipid stores, which have already been shown to include the bacterial food strain [6]. Therefore in order to compare the results between studies, many critical factors need to be considered.

Additionally, Soukas *et al.* normalized their data to total phospholipid amount and because both *rict-1* and *sgk-1* mutants are smaller than wild type, assuming that the change in size is due to a reduction in cell size and not cell number, one would expect the phospholipid amount to decrease per worm. This would result in a change in apparent triglyceride levels even if the total triglyceride levels were unchanged. Taken together, our data suggests that *rict-1* does not play a major role in fat accumulation in the developing worm, while *sgk-1* does.
