# Supplementary figures and images for: A Comparative Study of Fat Storage Quantitation in Nematode Caenorhabditis elegans Using Label and Label-Free Methods
Source: PLoS One. 2010 Sep 16;5(9):e12810. doi: 10.1371/journal.pone.0012810 (PMC2940797; doi:10.1371/journal.pone.0012810)

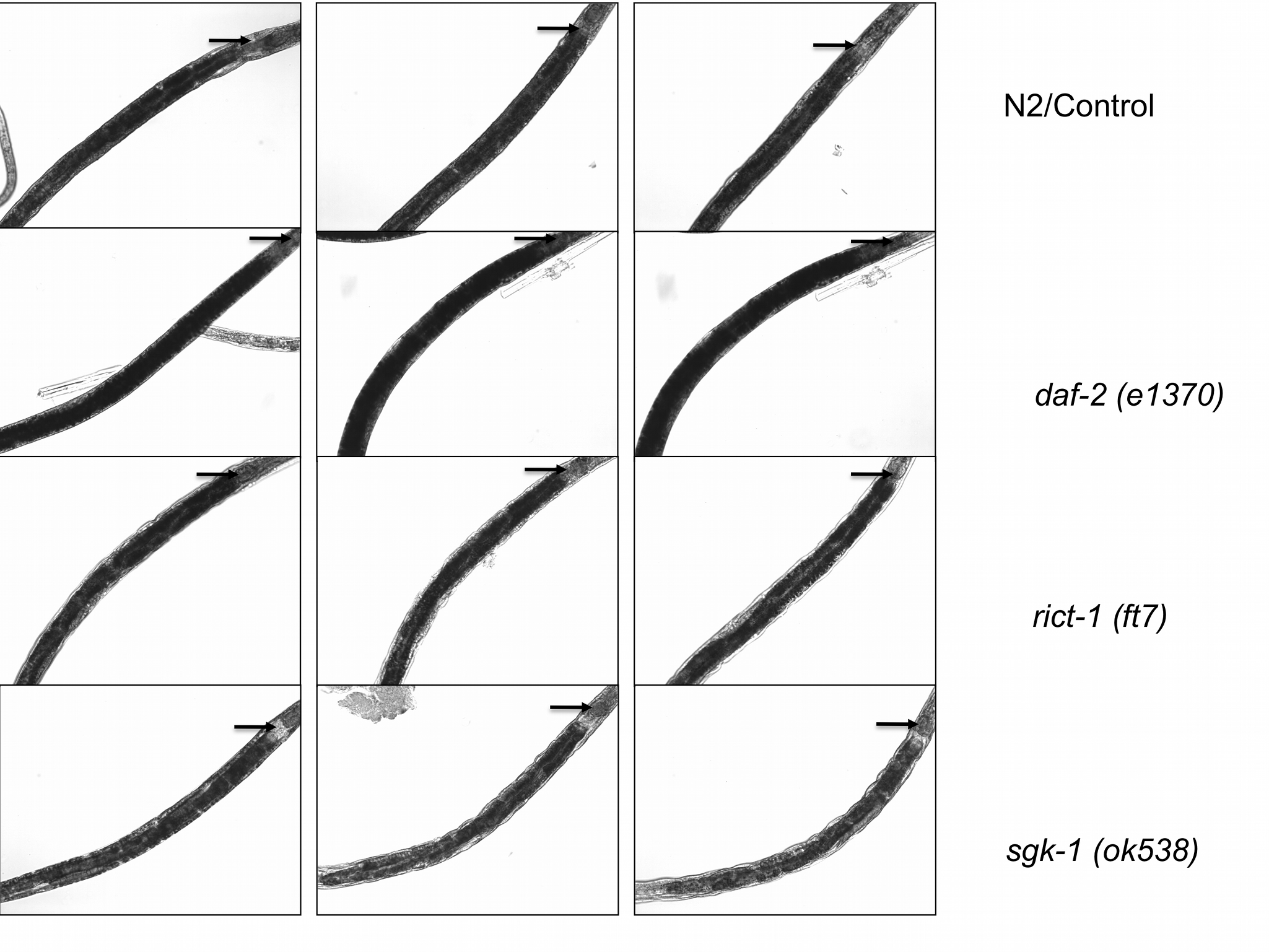

Supplement: Figure S1 — Additional Sudan Black stained worms. Arrow indicates the pharynx of the worm. (2.28 MB TIF) [file pone.0012810.s002.tif]

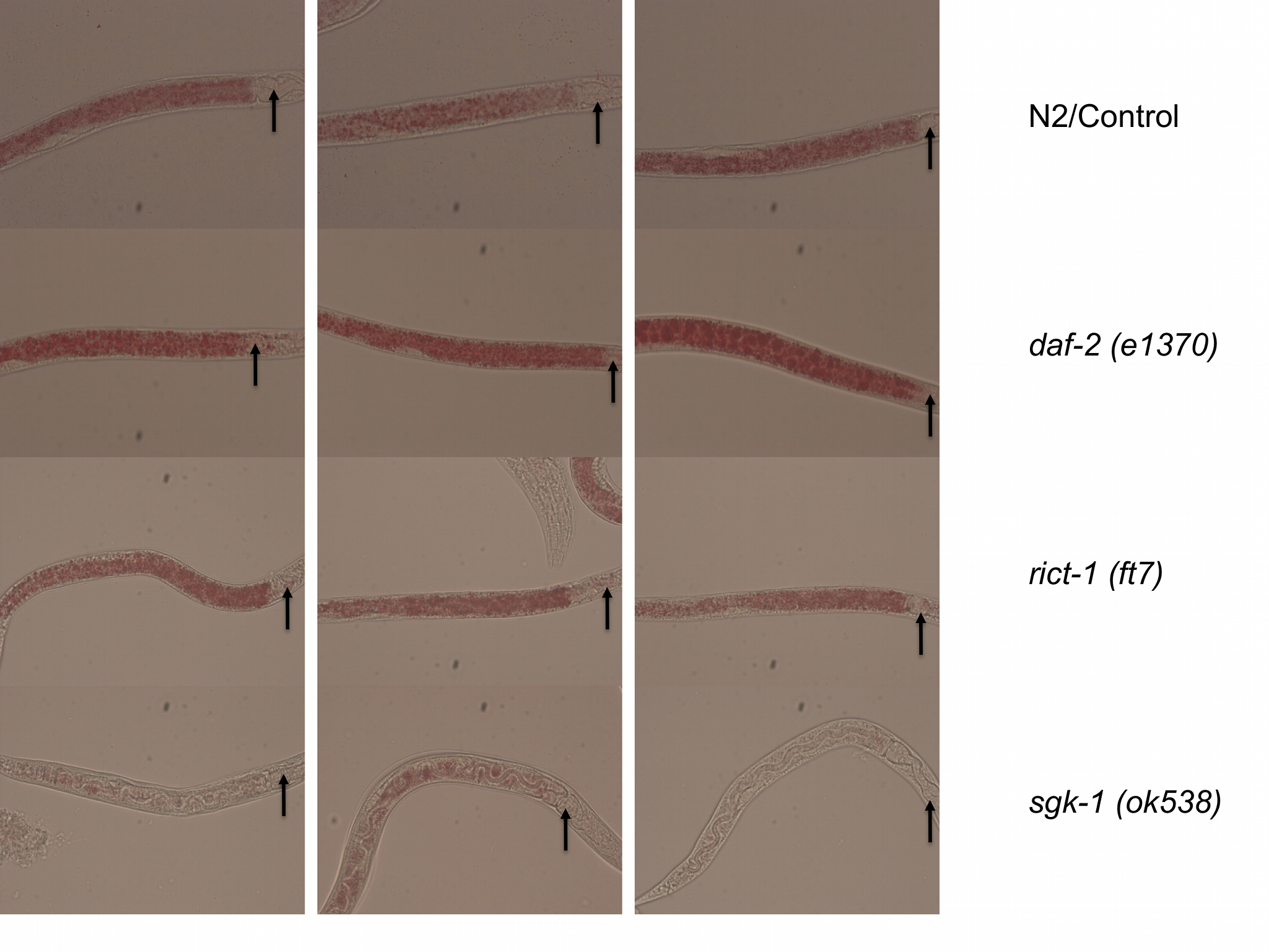

Supplement: Figure S2 — Additional Oil Red O stained worms. Arrow indicates the pharynx of the worm. (3.65 MB TIF) [file pone.0012810.s003.tif]

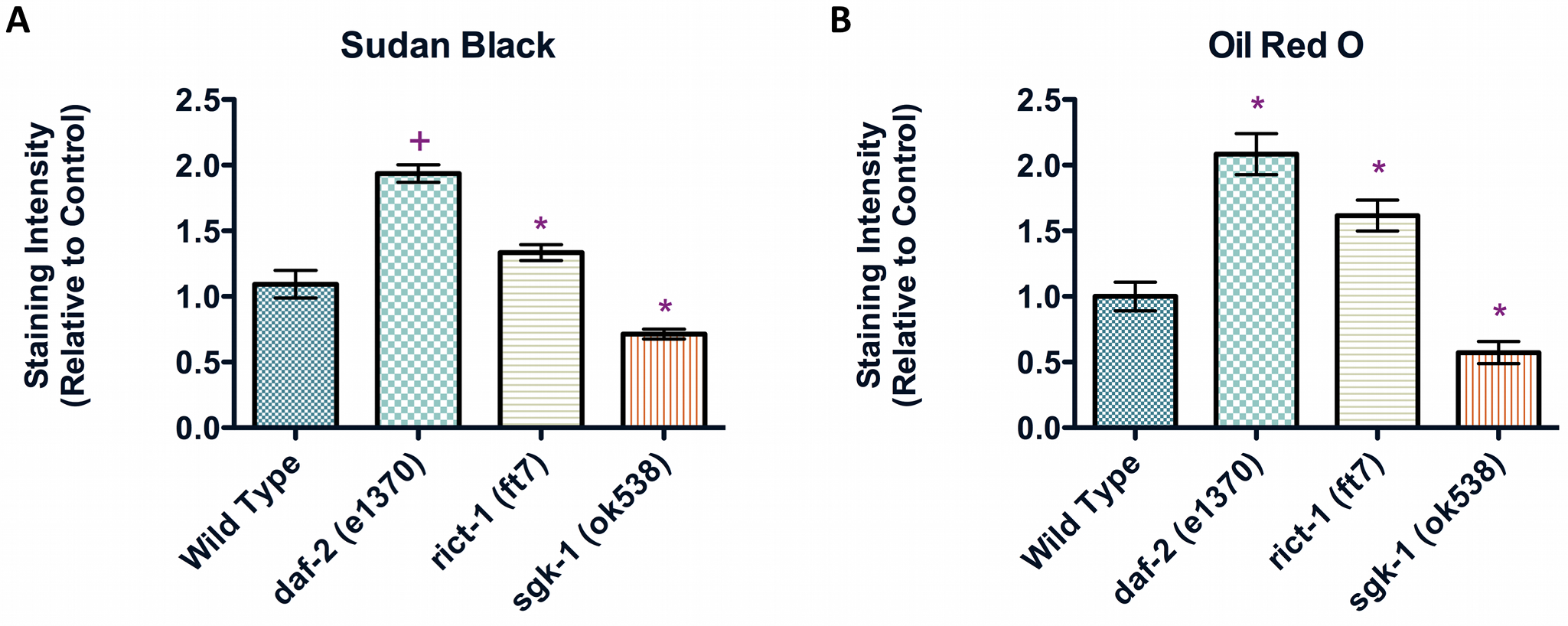

Supplement: Figure S3 — Quantification of Sudan Black and Oil Red O staining. Both daf-2 and rict-1 have increased staining compared to wild type. sgk-1 mutants have decreased staining. * indicates a significant difference compared to wild type. + in 2 out of 3 trials there was a significant difference from wild type. (1.44 MB TIF) [file pone.0012810.s004.tif]

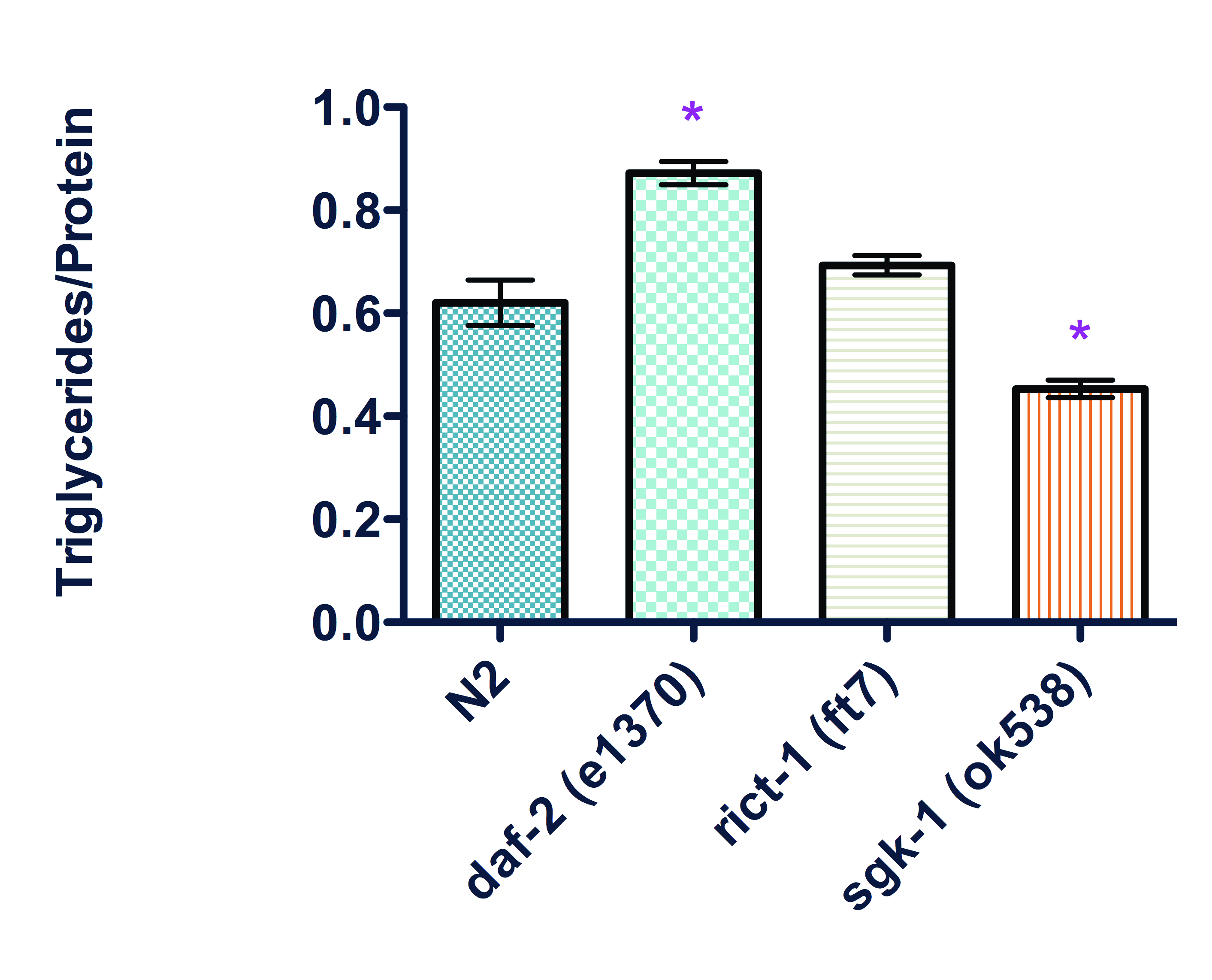

Supplement: Figure S4 — Quantification of triglyceride stores. Corroborating the data from the CARS quantification, there is a significant increase in triglycerides in daf-2 worms, a significant decrease in triglycerides in sgk-1 worms, and no change in triglycerides in rict-1 worms. Triglycerides has been normalized to total protein levels and the units for the Y-axis is nmoles of triglycerides/gram of protein. (0.49 MB TIF) [file pone.0012810.s005.tif]

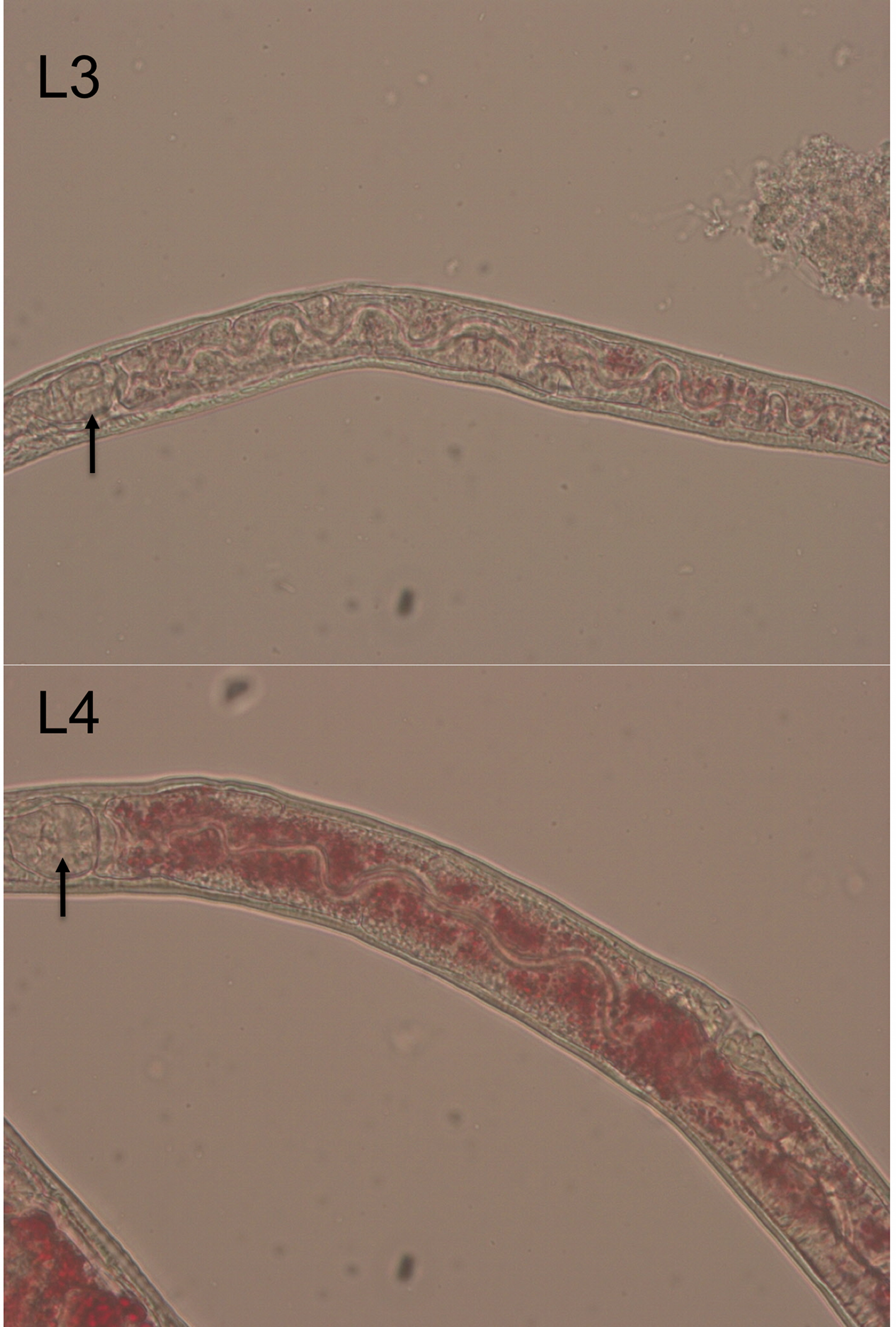

Supplement: Figure S5 — Differential fat stores in larval stage 3 (L3) worms compared to larval stage 4 (L4) worms. Typical Oil Red O staining of an L3 sgk-1 worm compared to an L4 sgk-1 worm. The top panel is an L3 worm and the bottom is an L4 worm. (7.78 MB TIF) [file pone.0012810.s006.tif]
